# Detection of *Alphitobius diaperinus* by Real-Time Polymerase Chain Reaction With a Single-Copy Gene Target

**DOI:** 10.3389/fvets.2022.718806

**Published:** 2022-03-09

**Authors:** Aline Marien, Hamza Sedefoglu, Benjamin Dubois, Julien Maljean, Frédéric Francis, Gilbert Berben, Stéphanie Guillet, Jean-François Morin, Olivier Fumière, Frédéric Debode

**Affiliations:** ^1^Quality and Authentication of Agricultural Products Unit, Knowledge and Valorization of Agricultural Products Department, Walloon Agricultural Research Centre, Gembloux, Belgium; ^2^Haute Ecole Louvain-en-Hainaut, Montignies-sur-Sambre, Belgium; ^3^Functional and Evolutionary Entomology, Gembloux Agro-Bio Tech, ULiège, Gembloux, Belgium; ^4^Eurofins Biologie Moléculaire France, Eurofins, Nantes, France; ^5^Biological Engineering Unit, Life Sciences Department, Walloon Agricultural Research Centre, Gembloux, Belgium

**Keywords:** insect, *Alphitobius diaperinus*, lesser mealworm, Coleoptera, detection, real-time PCR, cadherin, feed

## Abstract

Use of edible insects as an alternative source of proteins in food and feed is increasing. These last years, numerous companies in Europe have started producing insects for food and feed purposes. In the European Union, the use of edible insects for human consumption falls within Regulation (EU) No. 2015/2283 on novel foods. For feed, Commission Regulation (EU) 2017/893 authorizes seven insect species as processed animal proteins for aquaculture. Methods of authentication are required to check the conformity of the products. In this study, we propose a real-time polymerase chain reaction (PCR) method for the specific detection of the lesser mealworm (*Alphitobius diaperinus*), one of the species included in the shortlist of authorized insects. The selected target is the cadherin gene with a single-copy (per haploid genome) illustrated by our experimental evidence. The PCR test amplified a 134-bp fragment of the cadherin gene. The qualitative method was assessed toward several performance criteria. Specificity was checked against 54 insect species next to other animal and plant species. The sensitivity, efficiency, robustness, and transferability of the PCR assay were also successfully tested. Finally, the applicability of the test was assessed on real-life processed samples (industrial meals) of *A. diaperinus*. The study also showed that there seems to be a huge confusion on the correct labeling of the marketed mealworms. We did not succeed to get *Alphitobius laevigatus* samples. They all appeared to belong to the *A. diaperinus* taxon.

## Introduction

In recent years, edible insects are becoming an increasing alternative source of proteins. The main reason for it is that the food production rate is expected to be lower than the population growth (1). However, the insect consumption in European countries is limited because of strong impeding barriers associated with texture and appearance, much more than the taste (2, 3). Therefore, the incorporation of insects as ingredients in common food items such as sausages, protein bars, pâté, buns, and pastas is a way to overcome this problem (3).

Insects represent a promising strategy for enriching food in some nutrients, thereby achieving a better nutritional balance (4). In fact, the positive nutritional features of edible insects are the presence of high quantities of proteins, essential and non-essential amino acids, lipids, fibers, vitamins, and minerals (4, 5). Roncolini et al. (4) conducted a study to evaluate the use of lesser mealworm powder to replace a part of wheat flour as a means to enhance the protein and mineral content of crunchy snacks. The technological, microbiological, nutritional, and sensory characteristics of the fortified rusks were also evaluated in their study. A publication of Lacroix et al. (6) suggested the potential of lesser mealworm protein hydrolysates to serve as functional food ingredients to help improve glycemic regulation. Many insect species have been shown to have a high concentration not only of iron but also of zinc (3, 7). The rearing substrate can then modulate the insect nutritional quality (3). Insect growth rates, production efficiencies, and protein quality are influenced by substrates used to feed the insects but also depend on entomological species raised and rearing conditions (8, 9).

In animal production, the main protein source comes from either soybean (to feed cattle) or from fishmeal (to feed fish). However, soybean is associated with deforestation, use of genetically modified plant breeds, and massive use of pesticides, whereas industrial fishing to produce fishmeal depletes fish stocks (10). Insects are an ideal feed alternative because they are a good source of nutrients. Compared with conventional livestock, insects require less space for production (11), are expected to use less water (12), and emit less greenhouse gases (13), making them a more sustainable source of animal proteins (8).

Recently, seven insect species were authorized as processed animal proteins in aquafeed (14) and in pig and poultry feed (15): black soldier fly (*Hermetia illucens* L.), yellow mealworm (*Tenebrio molitor* L.), lesser mealworm (*Alphitobius diaperinus*), house cricket (*Acheta domesticus* L.), banded cricket [*Gryllodes sigillatus* (Walker)], field cricket (*Gryllus assimilis* F.), and common house fly (*Musca domestica* L.). Among these species, *A. diaperinus* can easily be reared to obtain protein flour for human consumption, as well as for feed material (16). In the meantime, the list of authorized insects as processed animal proteins was extended to silkworm (17).

Human consumption of edible insects in the European Union falls within Regulation (EU) No. 2015/2283 on novel foods (18). This means that food containing insects and their derived products must be subject to authorization once European Food Safety Authority (EFSA) has performed the risk assessments on their safety (4). In 2015, EFSA published a list of insect species potentially usable as food (19). Lesser mealworm (*A. diaperinus*) is included in this list. *T. molitor* larvae and *A. domesticus* are now assessed as safe novel food by EFSA (20–22), and dried larvae of the yellow mealworm are already authorized for placing on the market (23) with appropriate labeling of the food in which it is contained.

*A. diaperinus* is therefore used as feed for rearing animals and might be used for human consumption, once EFSA has given its approval for it as a safe novel food. A second species of the *Alphitobius* genus, *Alphitobius laevigatus* F., not listed by EFSA, is also marketed but it is only intended to feed house pets, including reptiles and amphibians.

Methods are required to check the conformity of these novel products. These last years, different methods of insect detection were developed. Mass spectrometry approaches were proposed for the detection of *T. molitor* and *G. assimilis* (24), *H. illucens* (25), *A. domesticus, T. molitor, Locusta migratoria* L., and *A. diaperinus* (25, 26), as well as the *Drosophila* genus (Fallén) (27). Mass spectrometry may also help to differentiate insect meals according to their taxonomic groups (28). However, an accurate identification by proteomic methods will only be achievable after more intensive sequencing efforts, given the obvious lack of proteomic data for insect species (24). An adapted sedimentation protocol concentrating the insect particles for their detection in feed by light microscopy was proposed by Veys and Baeten (29).

At present, real-time polymerase chain reaction (PCR) remains the reference technique for DNA detection in food or feed products (30–34). Therefore, detection methods by real-time PCR were also proposed for insects. Most PCR tests published were developed on mitochondrial DNA. This facilitates detection in processed food and feed in which the DNA may be degraded, as a mitochondrion contains several copies of its genome, and several mitochondria can be present in a single cell (35). This is applied for *H. illucens* detection in feed (36, 37), *Oxya chinensis* (Thunberg) in food (38), *Bombyx mori L*. in food (1) and feed (39), and *G. sigillatus* in food and feed (40). However, this multicopy characteristic is a disadvantage for quantitation purposes as the copy number per cell will be variable, depending on the considered tissue (36). This is why other publications focused on single-copy gene tests such as the real-time PCR test for *T. molitor* detection in food and feed (41) and *B. mori* in feed (39). In this study, we chose to focus on a chromosomal target that is a single copy per haploid genome.

This study proposes the first real-time PCR method for specific detection of lesser mealworm (*A. diaperinus*). The target used is the cadherin gene, which for *A. diaperinus* was characterized by Hua et al. (42). Its equivalent in *T. molitor* was already used as a target for the development of a real-time PCR test (41).

## Materials and Methods

### Samples

Insects were either collected in natural environment by trained entomologists, purchased from specialized companies, or provided by the Functional and Evolutionary Entomology laboratory of Gembloux Agro-Bio Tech (ULiège, Gembloux, Belgium). Insects were selected in order to cover several taxonomic groups or to have close relatives with a practical interest to the species considered. All insects were dead at the arrival at the laboratory. DNA extraction was mostly performed on a single individual, except for smaller insects for which several individuals were required.

Real-life processed samples (four industrial meals from different EU-based companies) of *A. diaperinus* were obtained through the International Producers of Insects for Food and Feed (IPIFF), but the origin of samples must remain anonymous.

For each *A. diaperinus* industrial meal, a mix containing 0.1% (in mass fraction) of *A. diaperinus* in a commercial fish feed (composition: fishmeal, fish oil, wheat gluten, protein concentrate extracted from pea, maize starch, yeast, lecithin, vitamins, and minerals) was prepared.

### DNA Extraction

Genomic DNA was extracted and purified from all samples following the CTAB-based method described in Annex A.3.1 of the international standard ISO 21571:2005 (43). Plasmid DNA was isolated from bacterial cultures with the help of the Genopure Plasmid Maxi Kit (Roche Diagnostics GmbH, Mannheim, Germany). The quality and quantity of DNA extracted from samples were estimated spectrophotometrically using a Nanodrop ND-1000 spectrophotometer at 260 nm (A260) and 280 nm (A280) absorbance. DNA purity was determined using the A260/A280 ratio. The amplifiability of the DNA extract was successfully checked by real-time PCR with the 18S target for insects (41), rbcL (44) for plants, and with a generic fish target (45) for fish. Other species were tested with targets developed or evaluated within the European Union Reference Laboratory for Animal Proteins in Feedingstuff^1^ (EURL-AP, 2017) (32) or with the 18S target (46, 47). Ten nanograms of DNA was used in the PCRs.

The industrial meals and the feed mixes at 0.1% of *A. diaperinus* were extracted following the method recommended by EURL-AP and based on the adaptation of the protocol of the Wizard Magnetic DNA Purification System for Food kit (Promega, Madison, WI, USA). This method is described in the EURL-AP Standard Operating Procedure^2^ (EURL-AP, 2014). The quantities tested for this purpose are also in line with the EURL-AP SOP.

### Primers and Probe for the Real-Time PCR

Eurogentec (Seraing, Belgium) synthesized the oligonucleotides. The primers and probe sequences developed for the detection of *A. diaperinus* are presented in Table 1. The probe for this latter method was labeled with the reporter dye FAM™ (6-carboxyfluorescein) at the 5′ end and the quencher dye TAMRA™ (tetramethyl-6-carboxyrhodamine) at the 3′ end. Table 1 also lists the primers used for the purpose of differentiating *A. diaperinus* from *A. laevigatus*.

**Table 1:** Primers and probe used within this study.

**Target**	**Name**	**Sequences 5^**′**^-3^**′**^**	**Amplicon size (bp)**
*Alphitobius diaperinus* specific cadherin target	Alphi-Dia-Cad-F	CCAAGTGACTCTCATCATTCAGGAT	134
	Alphi-Dia-Cad-R	CTGAAACCGTAATGTCTAGTTCACCTA	
	Alphi-Dia-Cad-P	FAM- CCATTGCAGATCCAAGTCCCCGAAA -TAMRA	
*A. diaperinus* large cadherin target	Alphi-Cad-Seq-F	GAAGTGCCTGATCCCAGTGC	208
	Alphi-Cad-Seq-R	TGAGTTCTGCTGTGTAAAGTGCG	
*A. diaperinus* COI targets	COI-Alphi-F	CGTAGATAAATTTACAGTTTATTGCC	760
	COI-Alphi-R	CAGGATGTCCAAAAAATCAAAATAA	
	COI-Alphi-F2	CAGGATTCGGAATAATTTCTCA	758
	COI-Alphi-R2	TGCAGGAGGAGTTCTTT	

### Real-Time PCR Method

Real-time PCR (total reaction volume of 25 μL) was performed on a Lightcycler 480 (Roche Diagnostics Ltd., Rotkreuz, Switzerland) thermocycler using the Universal Mastermix provided by Diagenode (Seraing, Belgium). The reaction mixture included 12.5 μL of Master Mix, 1.7 μL of each primer (5 μM), 1.5 μL of probe (9 μM), 2.6 μL of bidistilled water, and 5 μL of DNA and distributed on 96-well reaction plates (Roche Diagnostics) for specific thermocyclers. Wells were covered with an adhesive film and centrifuged (2 min at 500 revolutions/min) to eliminate any air bubble in the well-bottoms. The thermal program was applied as follows: 2 min at 50°C; 10 min at 95°C; 50 cycles of 15 s at 95°C and 1 min at 60°C.

### Specificity of the PCR Method

The specificity of the method was checked on 54 insect species of different taxonomic orders including 19 Coleoptera taxa other than *A. diaperinus*, 6 Diptera, 10 Orthoptera, 7 Hemiptera, 4 Hymenoptera, 5 Lepidoptera, 1 Neuroptera, and 1 Blattodea. One sample of larvae labeled buffalo worm and another sample of larvae considered as *A. laevigatus* were purchased from specialized companies. However, a Sanger sequencing trial (see Results) shows that these two samples belong to the *A. diaperinus* species and are therefore considered as such for the specificity test. The specificity tests were performed on two arachnids and six crustaceans, which like insects belong to the Arthropoda phylum, 1 mollusk and 33 species of vertebrates (12 terrestrial mammals, 6 sea mammals, 8 birds, 7 fish). The possibility of a cross-reaction with human DNA was also envisaged. Seven plant species frequently used in feed and food were included in the experimental setup (Table 2). Ten nanograms of DNA was used in the PCRs. Each DNA extract was tested at least in duplicate.

**Table 2:** Specificity of *Alphitobius diaperinus* PCR test on animal and plant species (*n* = 2).

**Taxonomic classification**		**Latin name or family**	**Common name**	**Origin**	**Results**
INSECTS	Coleoptera	*Alphitobius diaperinus* (Panzer)	Lesser mealworm	a	+ (m = 25.30, σ = 0.03)
		*Alphitobius diaperinus* (Panzer)	Lesser mealworm	b	+ (m = 31.66, σ = 0.01)
		*Alphitobius diaperinus* (Panzer)^*^	Lesser mealworm	b	+ (m = 32.23, σ = 0.02)
		*Alphitobius diaperinus* (Panzer)^**^	Lesser mealworm	b	+ (m = 29.11, σ = 0.02)
		*Pachnoda* sp. (Burmeister)	Dola's worm	b	–
		*Tenebrio molitor* L.	Mealworm	b	–
		*Zophobas morio* F.	Superworm	b	–
		*Carabus* sp. L.	Beetle	a	–
		Staphylinidae (Latreille)	Rove beetles	a	–
		Curculionidae/Scolytidae (Latreille)	True weevils	a	–
		Coccinellidae sp. (Latreille)	Ladybird	a	–
		Scarabidae sp. (Latreille)	Scarab beetles	a	–
		*Oxythyrea funesta* (Poda)	White-spotted rose beetle	a	–
		*Melolontha melolontha* L.	Cockchafer	c	–
		*Leptinotarsa decemlineata* (Say)	Colorado potato beetle	c	–
		*Cassida viridis* L.	Green tortoise beetle	c	–
		*Cicindela campestris* L.	Green tiger beetle	c	–
		*Nicrophorus humator* (Gleditsch)	Black sexton beetle	c	–
		*Nicrophorus vespillo* L.	Common burying beetle	c	–
		*Cetonia aurata* L.	Rose chafer	a	–
		*Lucanus cervus* L.^***^	Stag beetle	a	–
		*Rhynchophorus ferrugineus* (Olivier)	Red palm weevil	b	–
		*Cybister limbatus* F.	Diving beetle	b	–
	Diptera	*Hermetia illucens* L.	Black soldier fly	b	–
		*Tabanus* sp. L.	Horse fly	a	–
		*Sarcophaga carnaria* L.	Common fresh fly	c	–
		*Bombylius major* L.	Large bee-fly	c	–
		Syrphidae (Latreille)	Hover fly	a	–
		*Musca domestica* L.	House fly	a	–
	Orthoptera	*Locusta migratoria* L.	Migratory locust	c	–
		*Acheta domesticus* L.	House cricket	b	–
		*Gryllus bimaculatus* (De Geer).	Mediterranean field cricket	b	–
		*Gryllus assimilis* F.	Jamaican field cricket	b	–
		*Gryllus* sp. L.	Cricket	a	–
		*Locusta* sp. L.	Locust	b	–
		*Acheta* sp. L.	Cricket	a	–
		*Patanga succincta* (Johannson)	Bombay locust	b	–
		*Schistocerca* sp. (Forsskål)	Sold as ≪ small grasshopper ≫	b	–
		*Brachytrupes portentosus* (Lichtenstein)	Giant cricket	b	–
	Hemiptera	Aphididae (Latreille)	Aphid	a	–
		Anthocoridae (Fieber)	Bugs	a	–
		*Palomena prasina* L.	Green shield bug	a	–
		*Pyrrhocoris apterus* L.	Firebug	a	–
		Belostomatidae sp. (Leach)	Giant water bug	b	–
		*Psyllus* sp. (Latreille)	Jumping plant louse	a	–
		Cicadidae sp. (Latreille)	Cicada	b	–
	Hymenoptera	*Apis* sp. L.	Bee	a	–
		*Bombus terrestris* L.	Buff-tailed bumblebee	a	–
		*Vespula* sp. (Thomson)	Wasp	a	–
		*Oecophylla smaragdina* F.	Weaver ant	b	–
	Lepidoptera	*Biston betularia* L.	Peppered moth	a	–
		*Tineola* sp. (Latreille)	Moth	a	–
		*Bombyx mori* L.	Silkworm	b	–
		*Galleria mellonella* L.	Greater wax moth	a	–
		*Omphisa fuscidentalis* (Hampson)	Bamboo worm	b	–
	Neuroptera	*Chrysoperla carnea* (Stephens)	Green lacewing	a	–
	Blattodea	*Blatta orientalis* L.	Oriental cockroach	c	–
Arachnida		*Heterometrus longimanus* (Herbst)	Black scorpion		–
		*Haplopelma albostriatum* (Simon)	Tarantulas		–
Crustacean		*Euphausia superba* (Dana)	Antartic krill		–
		*Penaeus vannamei* (Boone)	Whiteleg shrimp		–
		*Crangon crangon* L.	Common shrimp		–
		*Nephrops norvegicus* L.	Langoustine		–
		*Homarus gammarus* L.	European lobster		–
		*Paralithodes camtschatieus* (Tilesius)	Red king crab		–
Mollusca		Teuthida sp. (Naef)	Squid		–
Terrestrial mammals		*Bos taurus* L.	Beef		–
		*Sus scrofa domesticus* (Erxleben).	Pork		–
		*Sus scrofa scrofa* L.	Wild boar		–
		*Ovis aries* L.	Sheep		–
		*Capra hircus* L.	Goat		–
		*Equus caballus* L.	Horse		–
		*Equus asinus* L.	Donkey		–
		*Lepus europaeus* (Pallas)	Hare		–
		*Capreolus capreolus* L.	Roe deer		–
		*Cervus elaphus* L.	Stag		–
		*Rattus rattus* L.	Rat		–
		*Homo sapiens* L.	Human		–
Sea mammals		*Stenella coeruleoalba* (Meyen)	Striped dolphin		–
		*Tursiops truncatus* (Montagu)	Bottlenose dolphin		–
		*Grampus griseus* (G. Cuvier)	Risso's dolphin		–
		*Ziphius cavirostris* (G. Cuvier)	Cuvier's beaked whale		–
		*Phocoena phocoena* L.	Harbor porpoise		–
		*Phocidae* (Gray)	Seals		–
Fish		*Salmo salar* L.	Salmon		–
		*Gadus morhua* L.	Atlantic cod		–
		*Scomber scombrus* L.	Atlantic mackerel		–
		*Clupea harengus* L.	Atlantic herring		–
		*Mallotus villosus* (Müller)	Capelin		–
		*Sprattus sprattus* L.	Sprat		–
		*Engraulis encrasicolus* L.	European anchovy		–
Birds		*Gallus gallus* L.	Chicken		–
		*Meleagris gallopavo* L.	Turkey		–
		*Numida meleagris* L.	Guinea fowl		–
		*Cairina moschata* L.	Muscovy duck		–
		Anser sp. L.	Goose		–
		*Coturnix japonica* (Temminck and Schlegel)	Quail		–
		*Struthio camelus* L.	Ostrich		–
		*Turdus merula* L.	Blackbird		–
Plants		*Glycine max* (Merr)	Soybean		–
		*Zea mays* L.	Maize		–
		*Brassica napus* L.	Rapeseed		–
		*Triticum aestivum* L.	Wheat		–
		*Oryza sativa* L.	Rice		–
		*Solanum lycopersicum* L.	Tomato		–
		*Beta vulgaris* L.	Sugar beet		–

*+ = Positive signal, – = No signal*.

* *Sample labeled buffalo worm was purchased from a specialized company but identified as A. diaperinus by Sanger sequencing*.

** *Sample labeled A. laevigatus was purchased from a specialized company but identified as A. diaperinus by Sanger sequencing*.

*** *The Lucanus cervus (protected species) was not collected in the environment but obtained from an old insect box coming from a private collection*.

*For positive samples, mean Cq values (m) and standard deviations (σ) are given in brackets. Origin of the insect samples is specified with “a” for insects collected by trained entomologists, “b” for insects purchased from specialized companies and “c” for the insects provided by the Functional and Evolutionary Entomology lab of Gembloux Agro-Bio Tech (ULiège, Gembloux, Belgium)*.

### Cloning of the Target, Copy Number Determination of the Plasmid DNA, and Dilutions

The 134-bp target from the *A. diaperinus* cadherin gene was ligated into the 3.9-kb pCR® 2.1-TOPO plasmid vector (Invitrogen, Merelbeke, Belgium) following the TOPO® TA Cloning® kit instructions (Invitrogen). Plasmid DNA isolated from bacterial cultures was linearized with the *Hin*dIII restriction enzyme (Promega) and then purified using phenol–chloroform–isoamyl alcohol.

The quantity of recovered plasmid DNA was converted into copy numbers as usual (36, 48, 49), taking into consideration that (i) 1 unit of absorbance at 260 nm corresponds to a concentration of 50 μg/mL of double-stranded DNA, and (ii) the mean molar weight of one base pair is set at 635 Da (50).

The sensitivity, efficiency, and robustness of the PCR test were determined on diluted plasmid DNA. These dilutions were performed in water until an estimated copy number of 10,000 copies/5 μL was reached. Higher dilutions of the target DNA were prepared in a solution containing 50 ng/μL of salmon sperm DNA as background DNA. Low binding tubes were chosen to minimize DNA losses.

### Copy Number Determination of *A. diaperinus* Genomic DNA and Dilutions

The quantity of genomic DNA corresponding to 36,000 target copies was estimated at 10.08 ng for *A. diaperinus* based on data from the animal genome size database (https://www.genome.size.com/results.php?page=3) at the University of Guelph (Ontario, Canada). The sensitivity (limit of detection [LOD]) and efficiency of the PCR test were determined on diluted genomic DNA. Genomic DNA dilutions were performed in a similar way as described for the cloned target.

### Limit of Detection

Target sensitivity was evaluated following the recommendations of the former AFNOR XP V03-020-2 standard (51). This standard no longer exists, but the principles detailed in it are still valid. The absolute LOD was determined for the PCR assay (primers + probe + amplification program) on dilutions of plasmid material and on dilutions of genomic material.

The subsequent dilutions had to contain 50, 20, 10, 5, 2, 1, and 0.1 copies of the target. Six PCRs had to be achieved for each dilution. The method's LOD_6_ was the smallest copy number for which the six PCRs were positive, but only if the highest dilution supposed to contain the 0.1 copy per reaction generated a maximum of one positive PCR signal on six replicates. If more than one positive signal was observed for the 0.1 copy, the DNA quantities had to be revised. The copy number corresponding to LOD_6_ was then tested 60 times on the same plate (determination of the LOD_95%_). The LOD_95%_ is validated as equal or below a given copy number if at least 95% of positive signals are recorded out of the 60 replicates. The highest acceptable copy number for LOD_6_ and LOD_95%_ is 20 copies.

### Efficiency

The efficiency of the PCR assay was calculated with a dilution series of genomic DNA and plasmid material at target levels of 5,000, 2,500, 1,000, 500, and 100 copies. Each dilution was analyzed in six replicates and on four runs. The efficiency has to be between 90 and 110% (52).

### Digital PCR

The number of copies of the nuclear and plasmid DNA dilutions at approximately 500 copies/5 μL (5 μL being the volume of the DNA extracts added in the real-time PCR mix) were checked by digital PCR. Digital PCR was performed on the Biomark™ HD system (Fluidigm Corporation, South San Francisco, CA, USA) using the 12.765 Digital Array™. These digital arrays comprise 12 panels (12 wells, thus 12 samples), each of which is partitioned into 765 individual PCR of 6 nL. The reaction mixture included 4 μL of Universal Master Mix with passive reference (Diagenode), 0.15 μL of each primer (18.1 μM), 0.15 μL of probe (28.8 μM), 0.4 μL GE sample loading reagent (Fluidigm), and 3.15 μL of plasmid DNA. Eight microliters of reaction mix was dispensed into each sample inlet, and ~4.6 μL of this reaction mix was distributed throughout the partitions within each panel using an automated NanoFlex IFC Controller (Fluidigm Corporation) (53). Two arrays were analyzed with for each, 11 replicates of plasmid dilution and one no template control. The thermal program was as follows: 10-min activation step at 95°C, 50 cycles of 15 s at 95°C for denaturation and 60 s at 60°C for annealing and extension.

The number of target molecules per panel was determined using the BioMark HD Digital PCR software.

### Robustness of the PCR Method

The method robustness was tested by introducing some slight deviations to the standard experimental conditions (54). Parameters considered were as usual (36, 52, 55): the annealing temperature (60 ± 1°C), the primer concentrations (standard or reduced by 30%), the probe concentration (standard or reduced by 30%), and the real-time PCR Master Mix volume (standard or ± 1 μL), which involves a final reaction volume of 25 ± 1 μL. Six replicates of the plasmid borne target at 20 copies/5 μL were tested in the conditions described in Table 3. The robustness was performed on two real-time PCR platforms: thermocycler Lightcycler 480 (Roche Diagnostics Ltd.) with Universal Mastermix by Diagenode and thermocycler QuantStudio™ 5 Real-Time PCR system (Applied Biosystems, Thermo Fisher Scientific, Foster City, CA, USA) with ABI TaqMan 2x Universal PCR Master Mix No AmpErase UNG (Applied Biosystems). The acceptance criterion is that all deviations to the standard protocol must give a positive result at a level of 20 copies of the target in the reaction (52).

**Table 3:** Experimental conditions tested to evaluate the robustness of the described *Alphitobius diaperinus* PCR test.

PCR machine	Lightcycler 480 (Roche Diagnostics Ltd) and QuantStudio 5 (Applied Biosystems)				
PCR reagent kit	Universal Mastermix (Diagenode s.a.) and ABI Taqman 2x Universal PCR Master mix (Applied Biosystems)				
Annealing temperature	59 and 61°C				
Primer concentration	Minus 30%	Standard	Standard	Standard	Standard
Probe concentration	Standard	Minus 30%	Standard	Standard	Standard
PCR volume	Standard (20 μL mix + 5 μL DNA)	Standard (20 μL mix + 5 μL DNA)	Standard (20 μL mix + 5 μL DNA)	Standard + 1 μL Mastermix (21 μL mix + 5 μL DNA)	Standard – 1 μL Mastermix (19 μL mix + 5 μL DNA)

### Applicability of the PCR Method

The applicability of the PCR method was checked in duplicate on four real-life processed samples (industrial meals) of *A. diaperinus* produced in the EU and on a fish feed containing 0.1% in mass fraction of *A. diaperinus* industrial meal. Two DNA extracts with two dilutions were tested by PCR in duplicate.

### Transferability of the PCR Method

The efficiency and the LOD (LOD_6_ and LOD_95%_) of the PCR assay were tested on genomic DNA in the laboratory of Eurofins Biologie Moléculaire France with conditions similar to those of the developer's laboratory (CRA-W, Gembloux, Belgium). The dilution series for the LOD test were carried out by this second laboratory starting from the solution at 500 copies/5 μL checked by digital PCR.

Real-time PCR was performed on thermocycler CFX96 Deep Well Real-time PCR Detection Systems (Bio-rad, Hercules, CA, USA) using the Applied Biosystems™ TaqMan™ Universal PCR Master Mix (Applied Biosystems). Reaction mixtures were distributed on Hard-Shell® 96-Well PCR Plates (Bio-rad) developed for the CFX96 thermocyclers. Wells were covered with adhesive film, and the plates were centrifuged to eliminate any air bubble in the well-bottoms.

### Amplicon Preparation for Sanger Sequencing

The PCRs to generate amplicons that had to be checked by Sanger sequencing were done as follows. For the large cadherin target, the PCR contained 1 μL of DNA extract from the sample to be checked, 6 μL of 5 × GoTaq® Flexi Buffer (Promega), 3 μL of 2 mM dNTP mix (Thermo Scientific, Waltham, MA, USA), 3 μL of bovine serum albumin (BSA; Roth, Karlsruhe, Germany), 1.8 μL of 25 mM MgCl_2_ solution (Promega), 3 μL of 5 μM forward and reverse primers, 0.15 μL of 5 U/μL GoTaq® G2 Flexi DNA Polymerase (Promega), and nuclease-free water to 30 μL. The thermal cycling conditions were set as follows: initial denaturation at 95°C for 5 min followed by 35 cycles at 95°C for 30 s, 56°C for 30 s, and 72°C for 1 min, and a final extension at 72°C for 10 min.

To generate amplicons focused on the cytochrome c oxidase subunit I (COI) targets (Table 1), PCRs were set up in at similar way and contained 1 μL of DNA extract from the sample to be checked, 6 μL of 5x GoTaq® Flexi Buffer (Promega), 3 μL of 2 mM dNTP mix (Thermo Scientific), 3 μL of BSA (Roth), 1.8 μL of 25 mM MgCl_2_ solution (Promega), 3 μL of 5 μM forward and reverse primers, 0.15 μL of 5 U/μL GoTaq® G2 Flexi DNA Polymerase (Promega), and nuclease-free water to 30 μL. The thermal cycling conditions consisted in an initial denaturation at 95°C for 5 min followed by 35 cycles at 95°C for 30 s, 47°C (COI_Alphi_F/R) or 52°C (COI_Alphi_F2/R2) for 30 s and 72°C for 1 min, and a final extension at 72°C for 10 min.

Five microliters of PCR products were run on a 1.2% agarose gel to check amplicon quality. The remaining 25 μL of PCR products was sent to Eurofins Genomics (Constance, Germany) for Sanger sequencing.

## Results

The cadherin gene of *A. diaperinus* was used to select a piece of DNA that is specific to the considered insect species. Appropriate primers and probe were designed to amplify a 134-bp fragment of the cadherin gene (Table 1). The latter is considered as a single-copy gene in several insect species such as the Lepidopteran *Ostrinia nubilalis* (Hübner) (56) or the Coleoptera *Diabrotica virgifera virgifera* (Le Conte) (57), which is an advantage for quantitation purposes.

The specificity was first investigated *in silico* using the Blast tool available on the National Center for Biotechnology Information (NCBI) database. The different blasts and the alignments of DNA sequences with other Coleopteran (58) performed indicated that the PCR test should be specific to the target species *A. diaperinus*. The alignment with the closest relative within the NCBI database is the *T. molitor* sequence. It shows some similarity but not enough for allowing primers and probes to hybridize (Supplementary File 1). Specificity was also experimentally tested on DNA from *A. diaperinus* of various origins as well as on 53 other non-target insect species, including 19 Coleoptera. Positive results were obtained only with all samples of *A. diaperinus*. No signal was obtained with the 53 other insect species, the 42 other animal species (arachnids, crustaceans, mollusk and vertebrates), and the seven plant species tested (Table 2).

A difficulty that appeared was to know if the test enables to distinguish *A. diaperinus* from *A. laevigatus*, a closely related species commercialized as feed product for non-farmed animals. Products labeled as *A. laevigatus* or as buffalo worm were tested, and a clear positive signal with the PCR test for *A. diaperinus* was observed. A doubt existed, however, if these samples really belonged to the *A. laevigatus* species. To check this hypothesis, Sanger sequencing was applied on PCR products of eight collected samples of *Alphitobius* (Supplementary File 2). Three targets were considered. The first one focused on a larger cadherin target (Table 1), which completely contains the smaller target of 134 bp. The two other targets focused on COI regions known as more variable and considered as suitable to allow a distinction between *A. diaperinus* and *A. laevigatus* according to published data. All Sanger sequencings were successful and showed identical sequences for the eight samples (Supplementary Files 2–5).

The sequences obtained for the cadherin target corresponded to the *A. diaperinus* sequence published by Hua et al. (2014-KC470207.1)^3^ (Supplementary File 3). The alignment of sequenced COI fragments showed that all analyzed specimens belong to the *A. diaperinus* species when compared with *A. diaperinus* and *A. laevigatus* reference sequences. The first COI portion corresponded to the *A. diaperinus* sequence published by Hong et al. (2020-NC_049092.1)^4^ (Supplementary File 4); this COI region is, however, not available for *A. laevigatus*. Reference sequences are available for both *Alphitobius* species for the second COI region considered. Results for that target showed that all samples of *Alphitobius* tested belonged to the *A. diaperinus* species (Supplementary File 5).

The amplification efficiency, LOD, and robustness were evaluated on plasmid DNA. The efficiency and LOD were also determined on genomic DNA.

To check that the number of copies in the dilutions used to assess the performance criteria was correct, the dilutions at approximately 500 copies/5 μL were estimated by digital PCR on a Biomark™ HD system. For the genomic DNA, the average obtained over the 10 measurements by digital PCR was 339 copies/5 μL with a variation coefficient at 9.45% (Table 4). This mean copy number measured was slightly lower than the expected value (based on the genome size of *A. diaperinus* and considering the cadherin gene as a single-copy gene). The difference between these values did not exceed a factor of 2, corresponding to the subsequent dilution. These results therefore confirm that the cadherin gene is a single-copy gene per haploid genome as mentioned in other studies (56, 57). However, in order to be closer to the expected values to evaluate the efficiency and LOD, new dilutions on the genomic DNA were carried out, taking into account the results obtained in digital PCR. On these new dilutions, the average obtained over the 22 measurements by digital PCR was 498 copies/5 μL with a variation coefficient at 9.21% (Table 5). The mean copy number measured was estimated at the expected value and it is from this dilution series that the efficiency and sensitivity were evaluated.

**Table 4:** Copy numbers obtained on dilution of genomic DNA at ~500 copies/5 μL by digital PCR on a Biomark™ HD system.

**Copy number of target/5 μL**	**Copy number mean of target/5 μL ± SD (σ)**	**Coefficient of variation**
343	339 ± 32.01	9.45%
357		
365		
396		
374		
349		
346		
329		
357		
271		

*Ten replicates were analyzed (n = 10)*.

**Table 5:** Copy numbers obtained on dilution slightly adapted of genomic DNA at ~500 copies/5 μL by digital PCR on a Biomark™ HD system.

**Array**	**Copy number of target/5 μL**	**Copy number mean of target/5 μL ± SD (σ)**	**Coefficient of variation**
1	523	498 ± 8.18	9.21%
	515		
	540		
	528		
	515		
	512		
	584		
	531		
	451		
	556		
	526		
2	501		
	401		
	479		
	526		
	445		
	470		
	470		
	470		
	484		
	526		
	404		

*Twenty-two replicates was analyzed on 12.765 Digital Array™ (n = 22)*.

The copy number of the plasmid material (linearized) was also checked, and the average copy obtained over the 22 measurements by digital PCR was 446 copies/5 μL with a variation coefficient at 9.26% (Table 6). The mean copy number measured was estimated at the expected value, and it is from this dilution series that the efficiency, sensitivity, and robustness were evaluated.

**Table 6:** Copy numbers obtained on dilution of plasmid DNA at ~500 copies/5 μL by digital PCR on a Biomark™ HD system.

**Array**	**Copy number of target/5 μL**	**Copy number mean of target/5 μL ± SD (σ)**	**Coefficient of variation**
1	443	446 ± 8.25	9.26%
	398		
	487		
	484		
	445		
	451		
	531		
	473		
	365		
	418		
	390		
2	434		
	421		
	493		
	448		
	432		
	487		
	434		
	451		
	473		
	476		
	374		

*Twenty-two replicates were analyzed on 12.765 Digital Array™ (n = 22)*.

The PCR efficiency was evaluated at 102.5% on plasmid DNA and 100.0% on genomic DNA. This was calculated, taking into account the mean Cq (quantification cycle) values obtained at the different copy numbers tested (from 5,000 to 10), and no outliers were encountered (Tables 7, 8). When calculated per plate, the efficiency was always higher than 90% and therefore met the acceptance criterion proposed by Broeders et al. (52).

**Table 7:** Cq values obtained on dilutions of plasmid material used for efficiency calculation and for LOD_95%_.

**Copy number of target**	**Cq (mean value) ± SD (σ) and (*n*)**
5,000	26.83 ± 0.06 (24)
2,500	27.85 ± 0.11 (24)
1,000	29.08 ± 0.09 (24)
500	30.08 ± 0.11 (24)
100	32.39 ± 0.23 (24)
10	36.14 ± 0.50 (60)

*For efficiency, each concentration was analyzed in six replicates and on four PCR plates (n = 24). For LOD_95%_, the concentration at 10 copies was analyzed in 60 replicates on 1 PCR plate (n = 60)*.

**Table 8:** Cq values obtained on dilutions of genomic material used for efficiency calculation and for LOD_95%_.

**Copy number of target**	**Cq (mean value) ± SD (σ) and (*n*)**
5,000	27.55 ± 0.07 (24)
2,500	28.60 ± 0.07 (24)
1,000	29.87 ± 0.09 (24)
500	30.89 ± 0.12 (24)
100	33.21 ± 0.19 (24)
10	36.78 ± 0.48 (60)

*For efficiency, each concentration was analyzed in six replicates and on four PCR plates (n = 24). For LOD_95%_, the concentration at 10 copies was analyzed in 60 replicates on 1 PCR plate (n = 60)*.

Concerning the sensitivity testing, the LOD_6_ was estimated at five copies following the AFNOR XP V03-020-2 standard approach (51) and also at five copies for the LOD_95%_ with 57/60 positive signals on plasmid DNA and 58/60 positive signals on genomic DNA. Sixty of 60 positive signals were obtained at the level of 10 copies with a mean Cq value of 36.14 cycles on plasmid DNA and 36.78 cycles on genomic DNA. Therefore, the PCR test easily reaches the recommended performance criterion (≤ 20 copies).

The PCR method robustness was also evaluated on plasmid DNA, with success. All tested deviations to the standard protocol delivered positive results at the level of 20 copies in the PCR.

Positive signals were obtained on industrial samples (PAPs of *A. diaperinus*) showing the applicability of the PCR test on real-life samples (Table 9).

**Table 9:** Mean Cq obtained with the *Alphitobius diaperinus* PCR test on processed samples from *A. diaperinus* and on mixes containing 0.1% in mass fraction of *A. diaperinus* in a commercial fish feed (*n* = 2).

**Identification of samples**			**Mean Cq obtained with** ***Alphitobius diaperinus*** **PCR test**	
			**Dilution 1×**	**Dilution 10×**
Pure industrial meals of *A. diaperinus* produced in the EU	n°1	Extract 1	21.38	24.87
		Extract 2	21.03	24.39
	n°2	Extract 1	19.69	23.17
		Extract 2	19.55	23.05
	n°3	Extract 1	23.65	27.09
		Extract 2	22.94	26.52
	n°4	Extract 1	22.52	26.04
		Extract 2	22.09	25.59
Fish feed containing 0.1% of *A. diaperinus* from the industrial meal n°1	Extract 1		32.62	35.59
	Extract 2		32.79	36.10
Fish feed containing 0.1% of *A. diaperinus* from the industrial meal n°2	Extract 1		29.97	33.53
	Extract 2		29.78	33.15
Fish feed containing 0.1% of *A. diaperinus* from the industrial meal n°3	Extract 1		33.13	36.38
	Extract 2		33.20	36.56
Fish feed containing 0.1% of *A. diaperinus* from the industrial meal n°4	Extract 1		32.74	36.41
	Extract 2		32.46	35.93

The applicability was tested on a commercial fish feed adulterated with a low content of processed *A. diaperinus*. The commercial fish feed was first tested as free of *A. diaperinus* and the amplifiability of the DNA extracts obtained from this fish feed was checked with a 18S rDNA target (46, 47).

The four mixes of fish feed containing 0.1% of different processed *A. diaperinus* meals were tested and gave positive results with the cadherin PCR test (Table 9). The 10-fold dilutions provided evidence that there was no inhibitory effect of the feed matrix on the amplification of the *A. diaperinus* target.

Concerning the transferability, the PCR efficiency was evaluated at 90.0% on genomic DNA in a second laboratory and therefore met the acceptance criterion proposed by Broeders et al. (52). Table 10 indicates the mean Cq values obtained with the different copy numbers tested (from 5,000 to 10), and no outliers were encountered. When calculated per plate, the efficiency was higher than 90% for three plates and slightly below for the fourth one with an efficiency at 87.7%. The LOD_6_ was estimated at five copies following the former AFNOR XP V03-020-2 standard approach (51) and at 10 copies for the LOD_95%_ with 60/60 positive signals. The mean Cq value at 10 copies is of 37.08 cycles. The PCR test easily reached the recommended performance criterion (≤ 20 copies). The transferability of the method was therefore demonstrated.

**Table 10:** Cq values obtained on dilutions of genomic material used for efficiency calculation and for LOD_95%_ for the transferability test.

**Copy number of target**	**Cq (mean value) ± SD (σ) and (*n*)**
5,000	27.26 ± 0.11 (24)
2,500	28.47 ± 0.16 (24)
1,000	29.92 ± 0.11 (24)
500	31.05 ± 0.14 (24)
100	33.37 ± 0.23 (24)
10	37.08 ± 0.80 (60)

*For efficiency, each concentration was analyzed in 6 replicates and on 4 PCR plates (n = 24). For LOD_95%_, the concentration at 10 copies was analyzed in 60 replicates on 1 PCR plate (n = 60)*.

## Discussion

The study describes a specific, sensitive, and robust test to detect *A. diaperinus*. With the recent authorization in the EU legislation to use eight insect species, among which lesser mealworm, in aquafeed, pig, and poultry feed, the interest of such a PCR test is increasing.

During the search for the target and the validation study, special care was taken to be able to distinguish lesser mealworm from other representatives of the *Tenebrionidae* family (Supplementary File 1). Blasting the sequences of the developed primers and of the targeted fragment against the NCBI nucleotide database showed that the PCR test is specific to the target species *A. diaperinus*. Unfortunately, cadherin gene sequences for other *Alphitobius* species are not available in the NCBI and DNA Data Bank of Japan databases. Sequence alignments were therefore not possible. The other *Alphitobius* species are, however, not produced nor marketed, with the exception of *A. laevigatus*.

It seems that there is an important confusion at commercial level between *A. diaperinus* and *A. laevigatus*. Indeed, *A. diaperinus* is known under the common names of lesser mealworm for the larvae (12, 14, 25, 42, 59) and darkling beetle at adult stage (19, 60, 61), whereas *A. laevigatus* is known as black fungus beetle^5^ (37, 62, 63). Especially as larvae, the name of buffalo worm can be used to designate both species (7, 9, 64–70). *A. laevigatus* looks like *A. diaperinus* in size and shape^3^ (62, 63). In a review on *Tenebrionidae* in France, Bonneau (71) also clearly points out that there are confusions between *A. diaperinus, A. laevigatus, Alphitobius piceus* Olivier, and *Alphitobius ovatus* Herbst.

This confusion seems also to occur at the level of the sequences available in the NCBI database. Two COI sequences published by (72, 73) (accession no. KM435102.1 and KM652640.1) attributed to *A. diaperinus* show 99% identity with *A. laevigatus* [KP410252.1, (74)] but only 88% with *A. diaperinus* [NC_049092.1, (75)].

This probably also explains that the ordered samples of *A. laevigatus* were wrongly labeled by their providers. The Sanger sequencing revealed that these samples were in reality *A. diaperinus* samples. No real commercial sample of *A. laevigatus* was found, and even if we did not check with all possible sources, it seems that it is not that easy to find marketed *A. laevigatus* larvae. That is the reason why from a merely practical point of view we consider that the 134-bp cadherin target published in this study is specific to *A. diaperinus*. Nevertheless, from a scientific viewpoint, it is not impossible that the target is specific only at genus level. It could not be checked because of a lack of appropriate samples to do so.

The applicability of the PCR method was also successfully tested on real-world samples. This validates the implementation of the method on industrial samples.

Finally, it should be stressed that the target is present only once per haploid genome, which makes it a suitable target for possible quantification purposes. This would require to have a general insect target, present only once per haploid genome. To our knowledge, such a target has unfortunately not yet been identified.

## Conclusions

The developed PCR method based on the cadherin gene fits for the purpose of detection of *A. diaperinus* in feed. Indeed, the efficiency met the required criterion, the specificity gave good results, and only *A. diaperinus* was detected with respect to the insect, animal, and plant species tested. The acceptance criteria were also reached for sensitivity (LOD_6_ and LOD_95%_) and robustness. The PCR method was applicable on real-life samples from industry even when *A. diaperinus* was present at 0.1% in mass fraction in a fish feed. Finally, the transferability of the method in a second laboratory was also demonstrated by testing the efficiency and LOD.

The developed method was primarily aimed for application on feed. However, it is not excluded that several of the PCR tests developed in the study might be helpful to taxonomists in a better delineation of the several taxons within the genus *Alphitobius*.

## Data Availability Statement

The original contributions presented in the study are included in the article/Supplementary Material, further inquiries can be directed to the corresponding author.

## Author Contributions

AM, HS, BD, and FD contributed to the design and implementation of the research. AM, HS, BD, JM, and FD designed and performed the experiments. SG and J-FM participated to the transferability study. AM, JM, BD, SG, J-FM, and FD analyzed the data and interpreted the results. AM wrote the manuscript with the help of FD. GB, BD, FF, SG, J-FM, and OF provided valuable comments to improve the quality of the manuscript. All authors contributed to the article and approved the submitted version.

## Funding

This research was financially supported by the European Commission in the frame of Horizon 2020 Public-Private Partnership Bio-Based Industries Joint Undertaking (topic BBI.2018.F2 – Large-scale production of proteins for food and feed applications from alternative, sustainable sources) through the FARMYNG project.

## Conflict of Interest

The authors declare that the research was conducted in the absence of any commercial or financial relationships that could be construed as a potential conflict of interest.

## Publisher's Note

All claims expressed in this article are solely those of the authors and do not necessarily represent those of their affiliated organizations, or those of the publisher, the editors and the reviewers. Any product that may be evaluated in this article, or claim that may be made by its manufacturer, is not guaranteed or endorsed by the publisher.

## Footnotes

^1^ www.eurl.craw.eu/wp-content/uploads/2021/01/EURL-AP-SOP-Ruminant-PCR-FINAL-V1.2.pdf

^2^ www.eurl.craw.eu/wp-content/uploads/2021/01/EURL-AP-SOP-DNA-extraction-V1.1.pdf

^3^ https://www.ncbi.nlm.nih.gov/nuccore/KC470207.1

^4^ https://www.ncbi.nlm.nih.gov/nuccore/NC_049092.1

^5^ https://www.ams.usda.gov/sites/default/files/media/StoredGrainInsectsReference2017.pdf
