# Supplementary material for: Detection of Alphitobius diaperinus by Real-Time Polymerase Chain Reaction With a Single-Copy Gene Target
Source: Front Vet Sci. 2022 Mar 9;9:718806. doi: 10.3389/fvets.2022.718806 (PMC8959938; doi:10.3389/fvets.2022.718806)
Supplement: Supplementary file 1 [file Data_Sheet_1.pdf]

Supplementary file 1. Alignment of the targeted cadherin gene portion of *Alphitobius diaperinus* with its equivalent in *Tenebrio molitor*. The location of the primers and probe Alphi-Dia-Cad is underligned.

```
KC470207.1_Alphitobius_diaperinus CCAAGTGACTCTCATCATTCAGGATATTGATGATAACATACCAGAGATCACTACGAAAGATGGCTCTAAA
DQ988044.2_Tenebrio_molitor       CACCATACTGCTGATCCTTGAAGACATTGATGACAGCATACCAGAAATCAGCGTACTTGATTCAGCCGAG
                                  *    *    ** *** ** * ** ******** * ********* ****        ***    *  *

KC470207.1_Alphitobius_diaperinus ---CCATTGCAGATCCAAGTCCCCGAAAACACTGCTGACTTAGGTGAACTAGACATTACGGTTTCAG
DQ988044.2_Tenebrio_molitor       AAGAAGATTGAAATCACTATCGATGAAG---GTGCTTCTGACGTGCCAGTCGACATCGTCGTCACCG
                                            * ***    **   ***     ****      *    * * *****    **  * *
```

Supplementary file 2. Samples of *Alphitobius* analysed in Sanger sequencing.

**Sample**	**Announced species**	**Origin of samples**
1	Buffalo worm	Larvae purchased from a specialized company
2	*A. laevigatus*	Larvae purchased from a specialized company
3	*A. diaperinus*	Insects collected by trained entomologists
4	*A. diaperinus*	Larvae purchased from a specialized company
5	*A. diaperinus*	Pure industrial meals produced in the EU n°1
6	*A. diaperinus*	Pure industrial meals produced in the EU n°2
7	*A. diaperinus*	Pure industrial meals produced in the EU n°3
8	*A. diaperinus*	Pure industrial meals produced in the EU n°4
