# Supplementary material for: Detection of Alphitobius diaperinus by Real-Time Polymerase Chain Reaction With a Single-Copy Gene Target
Source: Front Vet Sci. 2022 Mar 9;9:718806. doi: 10.3389/fvets.2022.718806 (PMC8959938; doi:10.3389/fvets.2022.718806)
Supplement: Supplementary file 2 [file Data_Sheet_2.PDF]

```
                   *      1180         *      1200         *      1220         *      1240         *      1260         *      1280         *      1300
KC470207.1   : GAAGTGCCTGATCCCAGTGCAAAAATAACAGACCAAGTGACTCTCATCATTCAGGATATTGATGATAACATACCAGAGATCACTACGAAAGATGGCTCTAAACCATTGCAGATCCAAGTCCCCGAAAACACTGCT
Sample1_ACS  : ------------CCCAGTGCAAAAATAACAGACCAAGTGACTCTCATCATTCAGGATATTGATGATAACATACCAGAGATCACTACGAAAGATGGCTCTAAACCATTGCAGATCCAAGTCCCCGAAAACACTGCT
Sample2_ACS  : ------------CCCAGTGCAAAAATAACAGACCAAGTGACTCTCATCATTCAGGATATTGATGATAACATACCAGAGATCACTACGAAAGATGGCTCTAAACCATTGCAGATCCAAGTCCCCGAAAACACTGCT
Sample3_ACS  : ------------CCCAGTGCAAAAATAACAGACCAAGTGACTCTCATCATTCAGGATATTGATGATAACATACCAGAGATCACTACGAAAGATGGCTCTAAACCATTGCAGATCCAAGTCCCCGAAAACACTGCT
Sample4_ACS  : ------------CCCAGTGCAAAAATAACAGACCAAGTGACTCTCATCATTCAGGATATTGATGATAACATACCAGAGATCACTACGAAAGATGGCTCTAAACCATTGCAGATCCAAGTCCCCGAAAACACTGCT
Sample5_ACS  : ------------CCCAGTGCAAAAATAACAGACCAAGTGACTCTCATCATTCAGGATATTGATGATAACATACCAGAGATCACTACGAAAGATGGCTCTAAACCATTGCAGATCCAAGTCCCCGAAAACACTGCT
Sample6_ACS  : ------------CCCAGTGCAAAAATAACAGACCAAGTGACTCTCATCATTCAGGATATTGATGATAACATACCAGAGATCACTACGAAAGATGGCTCTAAACCATTGCAGATCCAAGTCCCCGAAAACACTGCT
Sample7_ACS  : ------------CCCAGTGCAAAAATAACAGACCAAGTGACTCTCATCATTCAGGATATTGATGATAACATACCAGAGATCACTACGAAAGATGGCTCTAAACCATTGCAGATCCAAGTCCCCGAAAACACTGCT
Sample8_ACS  : ------------CCCAGTGCAAAAATAACAGACCAAGTGACTCTCATCATTCAGGATATTGATGATAACATACCAGAGATCACTACGAAAGATGGCTCTAAACCATTGCAGATCCAAGTCCCCGAAAACACTGCT

                        *      1320         *      1340         *      1360         *      1380         *      1400         *      1420
KC470207.1   : GACTTAGGTGAACTAGACATTACGGTTTCAGACATTGATACGG-----------------------------------------------GAGAAAACGCACTTTACACAGCAGAACTCA
Sample1_ACS  : GACTTAGGTGAACTAGACATTACGGTTTCAGACATTGATACGGTAAATACAACGACTTTTACCGCGGTTGTTTTATATCTGCTTTCTAGGGAGAAAACGCACTTTACACAG---------
Sample2_ACS  : GACTTAGGTGAACTAGACATTACGGTTTCAGACATTGATACGGTAAATACAACGACTTTTACCGCGGTTGTTTTATATCTGCTTTCTAGGGAGAAAACGCACTTTACACAG---------
Sample3_ACS  : GACTTAGGTGAACTAGACATTACGGTTTCAGACATTGATACGGTAAATACAACGACTTTTACCGCGGTTGTTTTATATCTGCTTTCTAGGGAGAAAACGCACTTTACACAG---------
Sample4_ACS  : GACTTAGGTGAACTAGACATTACGGTTTCAGACATTGATACGGTAAATACAACGACTTTTACCGCGGTTGTTTTATATCTGCTTTCTAGGGAGAAAACGCACTTTACACAG---------
Sample5_ACS  : GACTTAGGTGAACTAGACATTACGGTTTCAGACATTGATACGGTAAATACAACGACTTTTACCGCGGTTGTTTTATATCTGCTTTCTAGGGAGAAAACGCACTTTACACAG---------
Sample6_ACS  : GACTTAGGTGAACTAGACATTACGGTTTCAGACATTGATACGGTAAATACAACGACTTTTACCGCGGTTGTTTTATATCTGCTTTCTAGGGAGAAAACGCACTTTACACAG---------
Sample7_ACS  : GACTTAGGTGAACTAGACATTACGGTTTCAGACATTGATACGGTAAATACAACGACTTTTACCGCGGTTGTTTTATATCTGCTTTCTAGGGAGAAAACGCACTTTACACAG---------
Sample8_ACS  : GACTTAGGTGAACTAGACATTACGGTTTCAGACATTGATACGGTAAATACAACGACTTTTACCGCGGTTGTTTTATATCTGCTTTCTAGGGAGAAAACGCACTTTACACAG---------
```

Legend

- (green) : Alphi-cad-seq-F/Alphi-cad-seq-R binding sites
- (yellow) : Alphi-Dia-Cad-F/Alphi-Dia-Cad-R binding sites
- (cyan) : Alphi-Dia-Cad-P binding site

Supplementary file 3. Alignment of sequences of a portion of the cadherin gene. Alignment compares a sequence from *Alphitobius diaperinus* published by Hua et al. (2014 – KC470207.1) and the sequences obtained for 8 samples of *Alphitobius* collected on the market.
Note : The sequences obtained for the cadherin target correspond to the *A. diaperinus* sequence published by Hua *et al*. (2014 - KC470207.1). The alignment shows however a gap of 47 bases which must be due to the fact that the sequence published by Hua *et al*. was amplified from cDNA. Numbers above sequences indicate the position in the *A. diaperinus* cadherin sequence KC470207.1.
