# Supplementary figures and images for: Detection of Alphitobius diaperinus by Real-Time Polymerase Chain Reaction With a Single-Copy Gene Target
Source: Front Vet Sci. 2022 Mar 9;9:718806. doi: 10.3389/fvets.2022.718806 (PMC8959938; doi:10.3389/fvets.2022.718806)

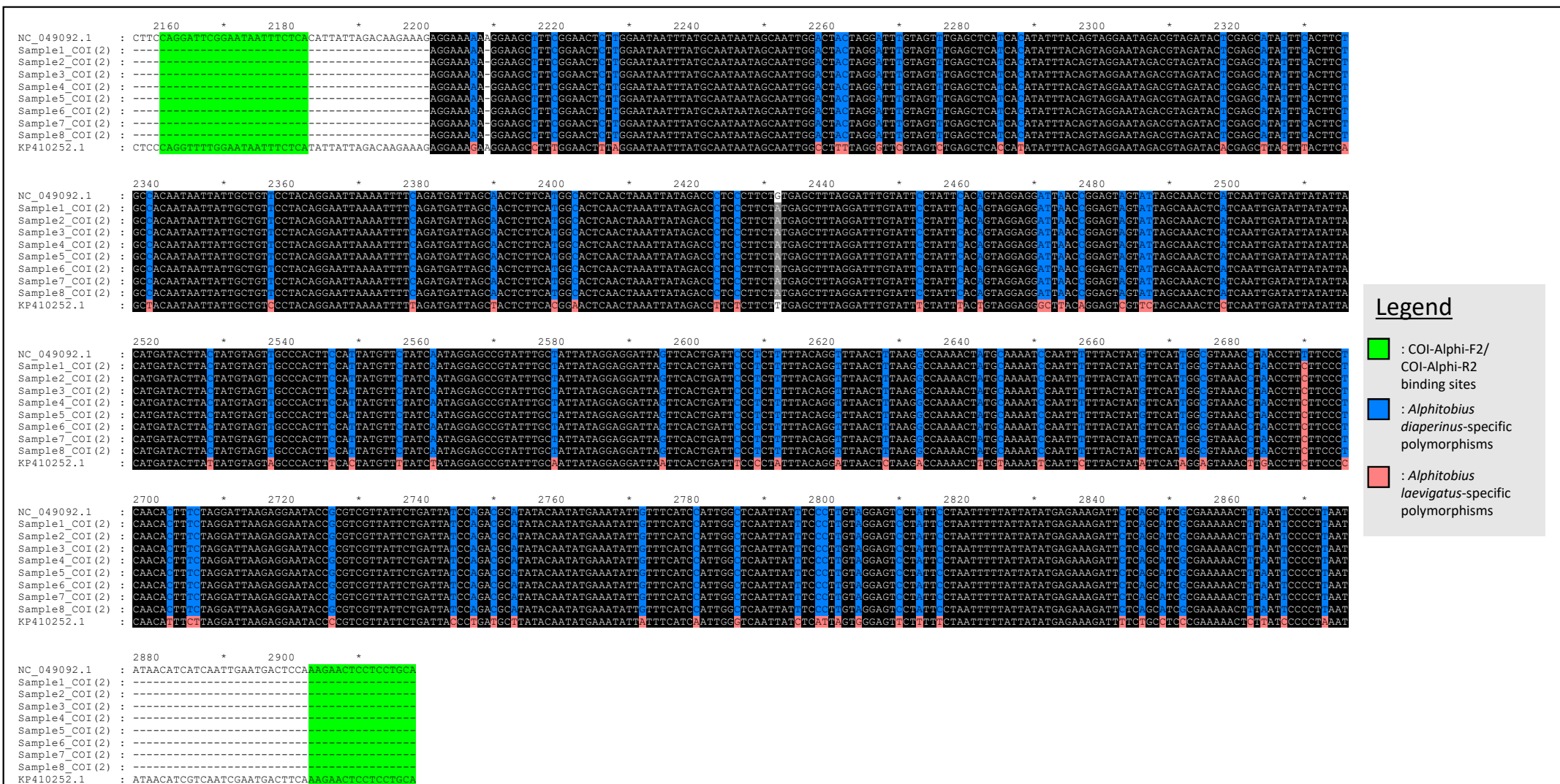

Supplement: Supplementary file 4 [file Data_Sheet_4.PDF]
