# Supplementary material for: Detection of Alphitobius diaperinus by Real-Time Polymerase Chain Reaction With a Single-Copy Gene Target
Source: Front Vet Sci. 2022 Mar 9;9:718806. doi: 10.3389/fvets.2022.718806 (PMC8959938; doi:10.3389/fvets.2022.718806)
Supplement: Supplementary file 3 [file Data_Sheet_3.PDF]

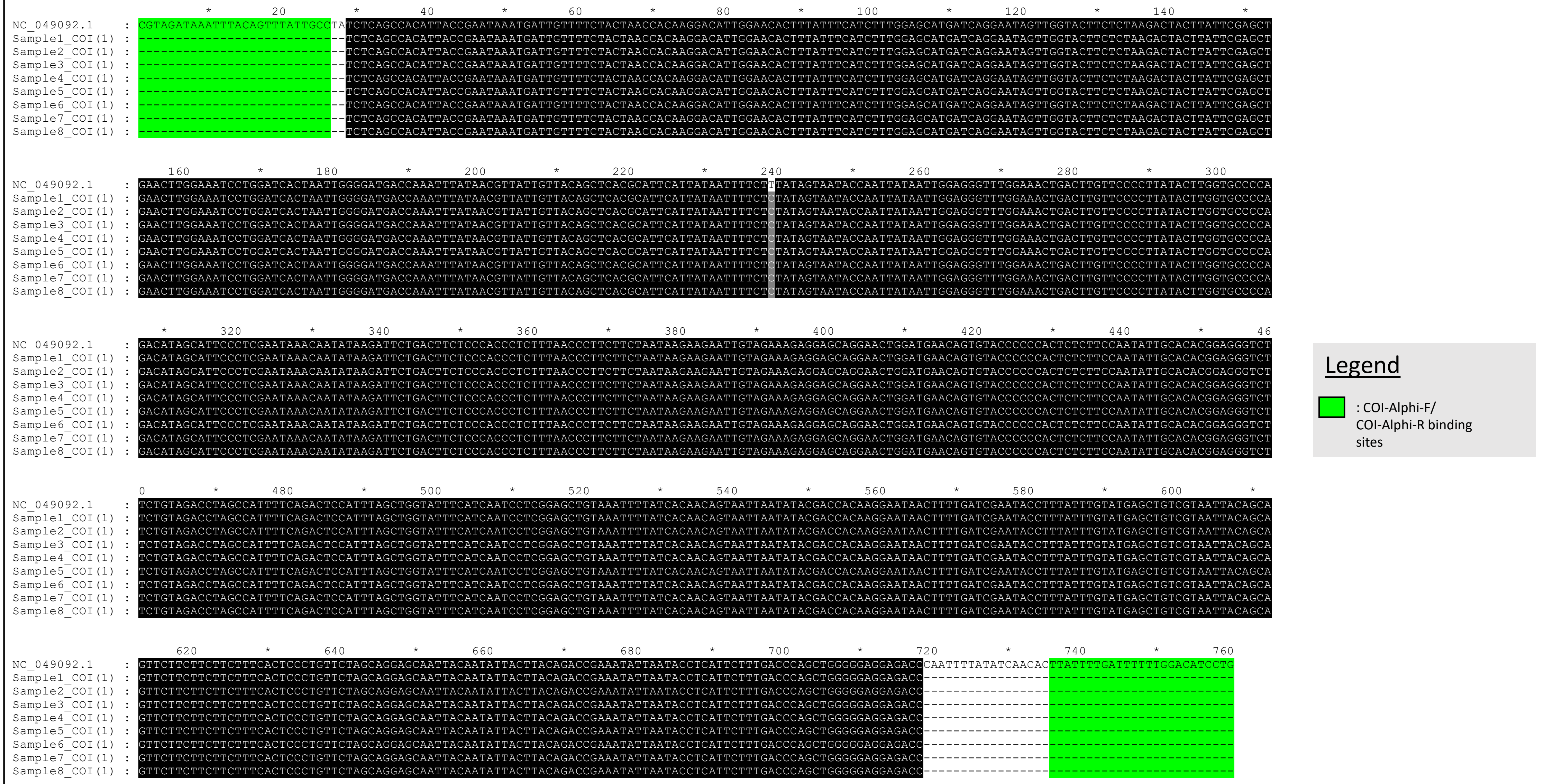

Supplementary file 4. Alignment of COI DNA fragments amplified with the COI-Alphi-F and COI-Alphi-R primers on the 8 samples of *Alphitobius* and the corresponding sequence extracted from the reference mitochondrial genome of *A. diaperinus* (NC_049092.1) published by Hong et al. (2020). Numbers above sequences indicate the position in the mitochondrial genome of *A. diaperinus* (NC_049092.1).
